# Complete genome sequence of *Staphylococcus aureus* strain MD04 isolated from the foot ulcer of a patient with diabetes in rural southwestern Uganda

**DOI:** 10.1128/mra.00362-25

**Published:** 2025-06-10

**Authors:** Danladi Makeri, Emmanuel Eilu, Martin Odoki, Ismail Abiola Adebayo, Reuben Maghembe, Samweli Bahati, Musoba Abubakar, Reagan Muhwezi, Theophilus Pius, Priscilla Peter Dilli, Saheed Adekunle Akinola, Ezera Agwu

**Affiliations:** 1 Department of Microbiology and Immunology, Kampala International University Western Campus, Ishaka, Uganda; 2 Department of Microbiology and Immunology, School of Medicine, King Ceasor University 442746 , Kampala, Uganda; 3 Department of Applied Sciences, School of Sciences, Nkumba University 183054, Entebbe, Uganda; 4 Department of Microbiology, Parasitology, and Immunology, School of Medicine, Kabale University 323124, Kabale, Uganda; 5 Omics and Bioinformatics Section, DABA Biotech Ltd, Dar es Salaam, Tanzania; 6 Department of Microbiology and Parasitology, Faculty of Medicine, St. Francis University College of Health and Allied Sciences 518753, Ifakara, Tanzania; 7 Department of Immunology and Molecular Biology, College of Health Sciences, Makerere University 58588, Kampala, Uganda; 8 Institute of Biomedical Research, Kampala International University Western Campus, Ishaka, Uganda; 9 Department of Medical Laboratory Science, Kampala International University Western Campus, Ishaka, Uganda; 10 Department of Public Health, Kampala International University Western Campus, Ishaka, Uganda; 11 Department of Microbiology and Parasitology, School of Medicine and Pharmacy, College of Medicine and Health Sciences, University of Rwanda 58621, Butare, Rwanda; Rochester Institute of Technology, Rochester, New York, USA

**Keywords:** wound bacteria, bacterial genomics, diabetic foot infection, WGS, Illumina sequencing, Uganda, diabetic foot ulcers

## Abstract

We report the whole-genome sequence of *Staphylococcus aureus* strain MD04, isolated from the foot ulcer of a diabetic patient in rural southwestern Uganda. The assembled genome is 2,789,538 base pairs in length, with a GC content of 32.5%. Genome completeness is 98%, with a 2.48% contamination.

## ANNOUNCEMENT

Diabetic foot ulcers (DFUs) are a major public health concern, especially in low-resource settings where access to specialized care is limited (1–3). They frequently become infected with multidrug-resistant pathogens such as *Staphylococcus aureus*, contributing to poor healing, limb amputation, and mortality (4–6). This genome announcement presents the draft genome sequence of *S. aureus* strain MD04, isolated from a chronic DFU in Uganda. It provides genomic insights into resistance and virulence genes to support genomic surveillance, antimicrobial stewardship, and pathogen evolution research.

Strain MD04 was isolated from a wound swab collected from a 58-year-old diabetic patient attending Kampala International University Teaching Hospital. The isolate was cultured on Mannitol salt agar (Himedia, India) and incubated aerobically at 37°C for 24 hours. Preliminary identification was based on Gram staining (gram-positive cocci clusters) and a positive catalase and coagulase test. Antimicrobial susceptibility testing was performed using the Kirby-Bauer disk diffusion method following CLSI guidelines (7), and it revealed resistance to multiple antibiotics, including cefoxitin, indicating methicillin resistance.

A single colony of the confirmed *Staphylococcus aureus* was grown overnight in Luria-Bertani broth (Himedia, India) in a shaking incubator at 37°C and 200 rpm, and cells were harvested by centrifugation at 12,000 × *g* for 10 min. Genomic DNA was extracted using the QIAamp DNA Mini Kit (QIAGEN, Germany), following the manufacturer’s protocol. The integrity of the extracted DNA was assessed by 1%, wt/vol agarose gel electrophoresis, and DNA concentration was measured using a Qubit dsDNA High Sensitivity Assay Kit (Thermo Fisher Scientific). DNA purity was evaluated using the NanoDrop ND-1000 spectrophotometer (Thermo Fisher Scientific). The whole genome sequencing was performed using the Illumina MiSeq platform with 2 × 150 bp paired-end reads. Library preparation followed the manufacturer’s instructions for the MiSeq DNA High Throughput Library Prep Kit (Illumina, USA).

Reads were quality-checked using FastQC (version 0.12.1) (8). Adapter sequences and low-quality bases were trimmed using Trimmomatic (version 0.39) (9), with a minimum read length cutoff of 36 bp. *De novo* assembly was performed with SPAdes (version 3.15.3) (10), resulting in 50 contigs. The genome was annotated using the NCBI Prokaryotic Genome Annotation Pipeline (11). The final assembled genome is 2,789,538 base pairs long, with a GC content of 32.5%, an estimated completeness of 98%, and a 2.48% contamination. It contains 2,756 genes, including 2,651 predicted protein-coding genes (Table 1). Default parameters were used for all software unless otherwise specified.

**TABLE 1:** Data availability, genome statistics, and features of *Staphylococcus aureus* MD04

Parameter	Value
Genome size	2,789,538 bp
Number of scaffolds	29
Scaffold *N*_50_	151.2 kb
Scaffold *L*_50_	6
Number of contigs	50
Contig *N*_50_	86.4 kb
Contig *L*_50_	10
GC%	32.5
Genome coverage	32.67×
Completeness (%)	98
Contamination (%)	2.48
Total number of genes	2,756

## Data Availability

This Whole Genome Shotgun project has been deposited in NCBI GenBank under the accession no. JBKICB000000000 (12). Raw reads are available in the NCBI SRA database under the accession number SRR31800042 (13). The annotated genome is accessible via the accession number ASM4657328v1 (14), and the associated BioSample is available under accession number SAMN45937523 (15).
